# Promoting Engagement With a Digital Health Intervention (HeLP-Diabetes) Using Email and Text Message Prompts: Mixed-Methods Study

**DOI:** 10.2196/ijmr.6952

**Published:** 2017-08-22

**Authors:** Ghadah Alkhaldi, Kerstin Modrow, Fiona Hamilton, Kingshuk Pal, Jamie Ross, Elizabeth Murray

**Affiliations:** ^1^ Community Health Sciences Department College of Applied Medical Sciences King Saud University Riyadh Saudi Arabia; ^2^ eHealth Unit Research Department of Primary Care and Population Health University College London London United Kingdom

**Keywords:** mHealth, eHealth, email, text messages

## Abstract

**Background:**

Engagement with digital health interventions (DHIs) may be regarded as a prerequisite for the intervention to achieve positive health or behavior change outcomes. One method employed to promote engagement is the use of prompts such as emails and text messages. However, little is known about the characteristics of prompts that promote engagement. This study explored the association between the content and delivery mode of prompts and the users’ engagement with HeLP-Diabetes (Healthy Living for People with type 2 Diabetes), a DHI that aimed to promote self-management in adults with type 2 diabetes.

**Objective:**

The objective of this study was to identify the characteristics of prompts, specifically the content and delivery mode, which were associated with increased engagement.

**Methods:**

This was a mixed-methods study. Email and text message prompts were sent to the registered users of HeLP-Diabetes. Use of the intervention was recorded and examined to identify which email and text message prompts were associated with subsequent visits to the DHI. Characteristics of prompts that were identified as particularly effective or ineffective were explored through think-aloud interviews with the participants.

**Results:**

Of a total of 39 email prompts, 49% (19/39) prompts showed a significant association with subsequent visits to the DHI. However, none of the text message prompts were associated with subsequent visits to the DHI. Furthermore, think-aloud interviews were carried out with 6 experienced participants with type 2 diabetes. The findings suggest that these participants preferred email prompts that were clear, relatively short, and empowering; used nondirective advice; included health professional references; were visually appealing; and contained news and updates.

**Conclusions:**

The findings of this study contribute to the existing evidence supporting the role of email prompts in promoting and maintaining engagement with DHIs. This study described the content of prompts that may be engaging. However, the results should be interpreted with caution, as prompts may be context-specific interventions and the results may not be generalizable across other DHIs or other types of interventions targeting self-management of type 2 diabetes.

## Introduction

### Importance of Digital Health Interventions

Digital health interventions (DHIs) are programs that provide emotional, decision, or behavior information and/or support for physical or mental health problems via digital platforms such as computers, mobile phones, or websites [1,2]. Lately, there has been a proliferation of evidence suggesting that DHIs may be effective in promoting self-care for chronic diseases, mental health, and health behavior change [1,3,4]. However, the effect sizes demonstrated in the systematic reviews of DHIs are often small [4-6], and this may be related to low engagement, or in some cases, lack of engagement with these DHIs [7-9].

### Importance of Engagement

It has been suggested that engagement, defined here as users’ regular interaction with part or all of the DHI [10], is positively associated with the effectiveness of DHIs, with a tendency toward a dose-response relationship [11-13]. Although the study of engagement is still in its infancy, there is a shared view that the promotion of engagement needs to be explored further [14].

### Importance of Prompts and Their Characteristics

One suggested method of promoting engagement is the use of technology-based prompts such as emails and text messages [8,15-17]. Several systematic reviews have shown that prompts are associated with positive engagement with DHIs [10,15,16], and one meta-analysis comparing the use of prompts against not using prompts showed that prompts have a small to moderate significant positive outcome (relative risk [RR] 1.27, 95% CI 1.01-1.60, I^2^=71%) [10]. However, the meta-analysis concluded that more research is needed to explore the differential effectiveness of the characteristics of various prompts, specifically their content and delivery modes [10].

There is relatively little work exploring whether the features of content and delivery are associated with enhancing engagement, and if so, identifying which features promote engagement. One study suggested that prompts with new content (eg, updated content on the DHI) were potentially associated with enhanced engagement [18]. A meta-regression that looked at the effect of text message and email prompts found both of these delivery modes to be effective in changing behavior [19].

### HeLP-Diabetes

HeLP-Diabetes (Healthy Living for People with type 2 Diabetes) is a DHI targeting self-management of type 2 diabetes developed by a research team from University College London (UCL). It was developed with a strong theoretical underpinning and following the principles of participatory design, where users were defined as patients with type 2 diabetes and health professionals’ caring for such patients. Focus groups conducted during the development process explored users’ views on engagement and the potential of prompts delivered via emails and text messages to promote engagement. In response to the focus group data [20] and the results of a systematic review that showed the potential of prompts in promoting engagement [10], emails and text messages were used to promote users’ engagement with HeLP-Diabetes. Prompts were sent to all the registered users 2 to 3 times per month.

HeLP-Diabetes was the subject of a research program that included two major studies undertaken in parallel: an individually randomized controlled trial (RCT) in primary care to determine effectiveness and cost-effectiveness [21] and an implementation study that aimed to explore how best to implement HeLP-Diabetes in National Health Service primary care practices [22]. The trial involved 20 primary care practices drawn from across England, with a mix of urban, suburban, and rural practices. The implementation study took place within one English clinical commissioning group (CCG) in London, the United Kingdom. Primary care practices in this area were excluded from the trial to avoid contamination. Participants were recruited to the trial between September 2013 and December 2014 and followed up for 12 months [21]. The implementation study took place between March 2013 and August 2015. The trial followed the standard “opt-in” recruitment procedures of adults aged 18 years or older who were registered with participating practices and had been diagnosed with type 2 diabetes. Participants were excluded if they were terminally ill, unable to use the intervention because of physical or mental impairment, or unable to provide informed consent. In the implementation study, the main outcomes were uptake and usage at the level of individual practices in the participating CCG. Practices were told that the intervention was available for use by any patient with type 2 diabetes and encouraged to refer all suitable patients to the program. In the implementation study, patients could use HeLP-Diabetes without participating in any research and were offered to use it as part of their routine diabetes care. Hence, the demographics of the user population differed between the two studies; in the implementation study, the demographics of users reflected the local population in that more than 50% came from black or minority ethnic backgrounds, one-third had only basic computer skills, and one-third had no formal education after minimum school leaving age. In contrast, the trial participants tended to be white (80%), and more than half of the participants rated themselves as experienced computer users. The study involved all the registered users of HeLP-Diabetes, that is, the trial participants who had been randomized to the intervention arm and all the patients registered through the implementation study.

### Aim and Objectives

In the eHealth (electronic health) and mHealth (mobile health) field, there has been a call to accelerate the pace of health research to correlate with the speed of technology development. One suggestion to accelerate eHealth research is to use studies with smaller samples that answer discrete, specific questions, as we aimed to do in this study, rather than conducting one major randomized controlled study [23]. Thus, this study was conducted with an overall aim to identify the characteristics of prompts, specifically the content and delivery mode, that had the potential to promote user engagement with HeLP-Diabetes. Specific objectives were as follows: (1) to identify prompts associated with increased numbers of subsequent visits to HeLP-Diabetes, (2) to identify prompts that appeared to have no association with numbers of subsequent visits to HeLP-Diabetes, and (3) to explore features of these selected prompts to understand why they did or did not appear to lead to subsequent visits to HeLP-Diabetes.

## Methods

### Study Design

**Figure 1:**
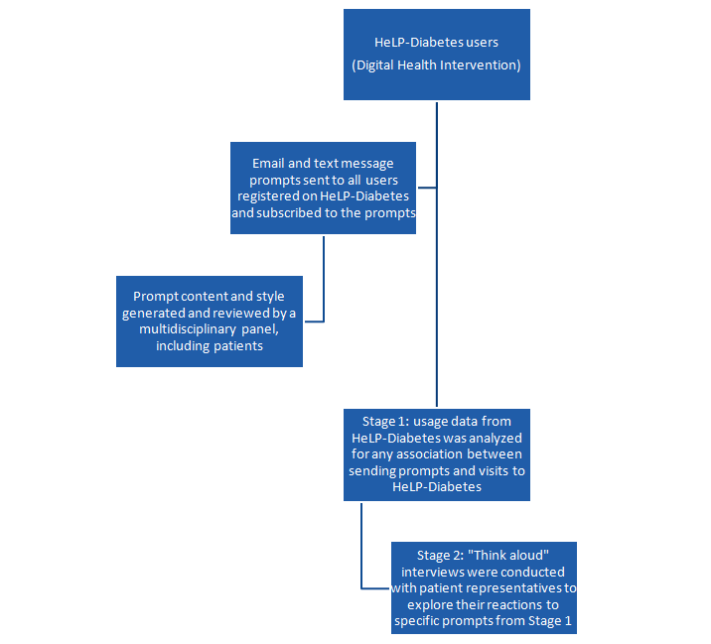
Flow diagram of study steps.

This was a mixed-methods study. It consisted of two components (see Figure 1): a quantitative component that analyzed usage data from the DHI to assess the association between sending email and text message prompts with subsequent visits to HeLP-Diabetes; and a qualitative component that comprised think-aloud interviews to explore user reactions to specific prompts that were selected based on the results of the quantitative component of the study. Ethical approval for the interviews was granted from UCL Ethics Committee (Project Identification number: 7263/001).

### Stage 1: Quantitative Component

Objective 1 was to identify prompts that were associated with increased numbers of subsequent visits to HeLP-Diabetes, whereas objective 2 was to identity prompts that appeared to have no association with the number of visits.

#### Participants

Participants included all the users registered on HeLP-Diabetes between March 2013 and May 2015 who had not specifically “unsubscribed” from receiving emails from the HeLP-Diabetes team. Hence, users were people with type 2 diabetes aged 18 years or older who were living in England, the United Kingdom. They had either volunteered to be a part of the RCT evaluating the clinical effectiveness and cost-effectiveness of HeLP-Diabetes or had been offered HeLP-Diabetes as a part of their routine care.

#### Design and Procedure

**Figure 2:**
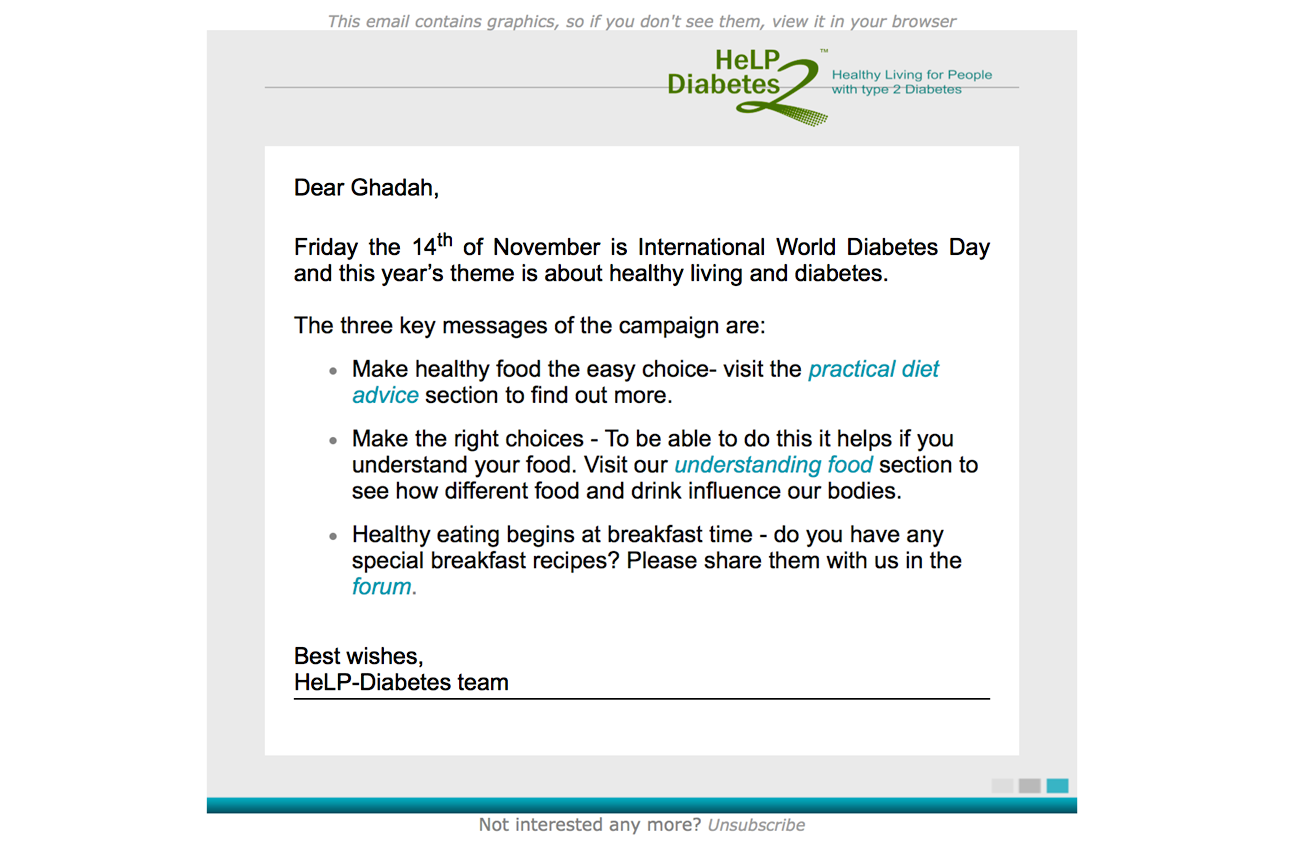
Example of email prompt “World Diabetes Day.”.

The primary goal of the email and text message prompts was to encourage users to visit the HeLP-Diabetes program; the prompts were not intended to have a direct impact on recipients’ self-care behaviors. Our proposed mechanism of action was that the prompts would bring key areas of the HeLP-Diabetes program to the attention of users and hence encourage visits. The HeLP-Diabetes program was considered the “active ingredient” in terms of promoting self-management. Content for prompts was developed through discussions with a multidisciplinary panel that included patient representatives, general practitioners, diabetes nurses, psychologists, dietitians, and project managers. Prompts were written by the lead author (GA) every month, with content selected to reflect recent diabetes-related news or research, HeLP-Diabetes updates, seasonal events, and other contemporary topics. In general, prompts opened by greeting the users with their username, followed by an introduction to the topics covered in the prompt and links to the relevant parts of HeLP-Diabetes. They ended with the HeLP-Diabetes contact email and the option of unsubscribing from future emails. Due to word count limits, text messages were usually limited to an introduction to the topic of the prompts and the relevant link (see Multimedia Appendices 1 and 2 for prompts content and examples, and Figure 2 for an example of an email prompt). The draft prompts were circulated to the multidisciplinary panel for feedback on content and tone and proofread before being sent to HeLP-Diabetes users. Email prompts were sent using the program “Acymail,” which enabled tracking which emails were opened and by how many users (“email open” rates).

The initial frequency of prompts was based on the advice from the participatory design panel and revised in line with subsequent feedback from patients’ representatives. Initially, the frequency for the prompts was 1 prompt per week; it was subsequently changed to 3 prompts per month. The prompts were not automated, hence a researcher was assigned to send them when they were finalized as per the abovementioned procedure; this resulted in differences in time periods between prompts. Any new user registering would only receive the prompts sent after their registration date. When users registered on HeLP-Diabetes, they were automatically subscribed to emails and given the option to subscribe to text messages. Email prompts were first sent in November 2013, whereas text message prompts started from October 2014. Recipients could unsubscribe from emails and/or text messages at any time.

#### Outcome Measures

Usage data (website metrics) refer to parameters that are constructed from digital traces left by users of digital interventions [24]. The usage data for this study included the number, date, and time of visits to HeLP-Diabetes, number of users to whom prompts were sent, users’ identification numbers, dates and numbers of emails and text messages sent, and dates and number of email opens. Usage data were collected for both types of prompts; for email prompts, data were collected between February 2014 and May 2015, and for text messages, between October 2014 and May 2015 (see Multimedia Appendix 1 for list of prompts content). The usage data used in this stage were downloaded from HeLP-Diabetes. Usage data were recorded on HeLP-Diabetes and calculated from server logs that recorded pages viewed by user identification numbers for the duration of the study period. We planned to use Google Analytics for collecting usage data initially, but pilot work showed that the data quality was poor and not all user activity was being appropriately recorded.

##### Primary Outcome Measure

###### HeLP-Diabetes User Visits

“HeLP-Diabetes user visits” referred to the user logging into HeLP-Diabetes in the period between prompts. The HeLP-Diabetes user visits measure was used for both email and text message prompts. This was a binary outcome and not a continuous measure—people either visited or they did not in each period, and multiple logins were not counted as multiple visits. For example, if a user visited HeLP-Diabetes after receiving an email prompt twice before the next prompt was sent, only 1 visit was counted for that user.

##### Secondary Outcome Measure

###### Email Opens

The “email opens” measure was used for email prompts only. An email was counted as opened if the user’s email client (eg, Yahoo Mail, Gmail, Outlook) downloaded the images embedded in the email, as this is the industry standard [25]. However, it is not a completely accurate measure because if images were blocked by email clients, users may be able to read text content without being counted as opening the email. Therefore, the reported email opens numbers may be an underestimate of the actual ones. Additionally, this measure only stored the details of the last time the email was opened; for example, if a user opened an email on the day he/she received it and subsequently reopened it, only the latter open was recorded.

#### Analysis

##### Email Prompt Analysis

Statistical analysis was conducted using the Statistical Package for the Social Sciences (SPSS) version 22. To describe the time over which prompts appeared to be associated with subsequent visits, we analyzed the data to identify the median and interquartile range (IQR) for the number of days between sending an email prompt and subsequent visits. This analysis suggested that any association of the prompts was limited to N days, and all subsequent analyses were limited to the visits that occurred within N days of an email prompt being sent. Thereafter, the association between opening an email prompt and visiting HeLP-Diabetes for each prompt within N days was analyzed with the chi-square test, using an alpha of <.05 to indicate statistical significance. A group of email prompts that showed a significant association between opening a prompt and visiting HeLP-Diabetes had a mixture of low and high number of visits, and those that did not show an association were selected visually to be explored in the think-aloud interviews in stage 2.

##### Text Message Prompt Analysis

Chi-square test was used to find any significant association between sending a text message prompt and visiting HeLP-Diabetes before the next prompt was sent (whether an email or a text message prompt). An alpha of <.05 was used to indicate statistical significance

### Stage 2: Qualitative Component

Objective 3 was to explore the features of specific prompts to understand why they did or did not appear to lead to subsequent visits to HeLP-Diabetes.

#### Participants and Procedure

The sample was a convenience sample, as participants were 6 patient representatives who had been involved in the development of HeLP-Diabetes and the prompts. They were invited to participate in the think-aloud interviews via email. The interviews took place at a time convenient to the participant and at the UCL eHealth unit. During the interview, participants were introduced to think-aloud interviews, and they were encouraged to say all their thoughts and opinions about each prompt, irrespective of whether their thoughts or opinions were negative or considered by them to be insignificant. A short practice was performed at the beginning of the interview to familiarize the participants with the think-aloud techniques (eg, speaking their first thoughts loudly). They were then asked to choose one of the email prompts that had not shown a significant association between opening it and visiting HeLP-Diabetes in stage 1 of the study to practice on. They viewed the prompts on a computer screen. After that, participants were randomly shown the email prompts that the stage 1 of the study had found to be significantly associated with visiting HeLP-Diabetes. They were asked to vocalize their thoughts and opinions while opening each email and describe what they liked or disliked about the content of each prompt and their first impressions or thoughts. After viewing all the email prompts, they were asked some questions based on what they expressed while viewing the emails, and what other participants had expressed in previous interviews (the interview guide evolved throughout the interviews). The interviewer (GA) took notes while the participants viewed the emails and prompted them when they forgot to vocalize their thoughts aloud. At the end of the interview, GA asked the participants for some basic demographic information, namely their age, sex, highest level of education achieved, how long they had had diabetes for, and how they rated their expertise with computers (basic, intermediate, or advanced), as these characteristics may influence participants’ perceptions of email prompts [26-28]. Once the participants finished their session, they were thanked and provided with a £20 voucher in appreciation for their help and reimbursed for any travel expenses. This stage of the study took place between July and September 2015 (see Multimedia Appendices 2 and 3 for the email prompts used in the think-aloud interviews and the interview schedule guide, respectively).

#### Analysis

Interviews were recorded and anonymized. They were transcribed verbatim by a professional and discreet transcriber who had signed a confidentiality agreement. Each participant had an identification number to ensure their anonymity (eg, P1). NVivo 10 was used for data management and analysis. An inductive thematic analysis approach was used. This started with familiarization with the transcripts and any notes taken during the interviews, followed by identification of themes that were strongly linked to the data rather than using preconceptions or a preexisting coding frame. An open coding process was applied where the transcripts were coded line-by-line and paragraph-by-paragraph. In addition, an “in vivo” coding (ie, coding using participants’ words) and constant comparative method, where we constantly compared the data across all the interviews by moving back and forth between them, were used. Emerging codes and themes were discussed and presented within the HeLP-Diabetes team meetings to ensure rigor and thoroughness of the analysis and to include expert and multidisciplinary input in the interpretation of findings.

#### Patient Involvement

Patients with type 2 diabetes who were involved in the development of HeLP-Diabetes were also involved in the development of the prompts. These patient representatives provided feedback and suggestions on topics, content, and the frequency of prompts. They also participated in the think-aloud interviews.

## Results

### Overview

The results are divided into two sections. The first section presents the results of the analysis of the email and text message prompts, whereas the second section presents the results of the think-aloud interviews with participants exploring the content of email prompts.

### Stage 1

Objective 1 was to identify prompts that were associated with increased numbers of subsequent visits to HeLP-Diabetes, whereas objective 2 was to identify prompts that appeared to have no association with the numbers of visits.

#### Email and Text Message Prompts’ Characteristics

Between February 2014 and May 2015, 49 prompts were sent to all registered HeLP-Diabetes users; of these, 42 were email prompts and 7 were text message prompts. The number of users who received the prompts ranged between 69 (for prompts sent early in the study) and 432 (for later prompts). The period between each prompt and the next ranged from 3 to 24 days (see Multimedia Appendix 1 for prompts’ date, delivery mode, content, number of recipients).

#### Participants’ Characteristics

The number of patients registered to use HeLP-Diabetes increased steadily throughout the study period. Each of the prompt recipients had different percentages of characteristics. For example, of the 411 users with available characteristics information who were sent “HeLP Diabetes Newsletter 20-What can you eat?,” 60% (247/411) were male, 41.3% (170/411) were aged between 41 and 60 years, and 51.3% (211/411) were aged 61 years or older. Furthermore, 18% (74/411) participants had had diabetes for less than a year, 33.8% (139/411) for 1 to 5 years, 19.7% (81/411) for 5 to 10 years, 21.6% (89/411) for 10 to 20 years, and the rest for over 20 years 5.8% (24/411) or not stated 0.9% (4/411). Of the 200 people who answered the question about previous computer experience, 38% (76/200) described it as “basic,” 37% (74/200) as “intermediate,” and 25% (50/200) as “advanced.”

#### Association Between Email Prompts and Visits to HeLP-Diabetes

**Figure 3:**
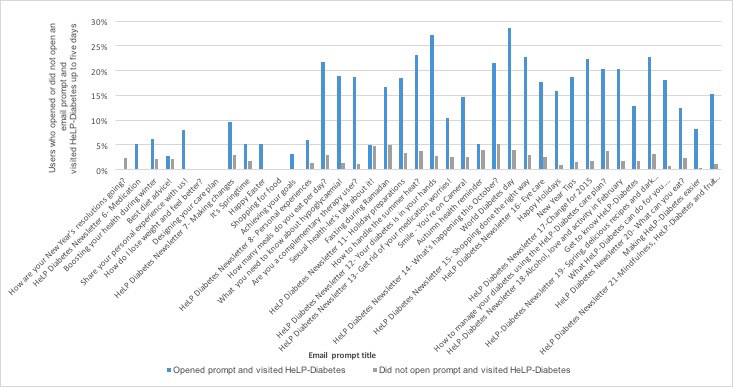
Percentage of users who opened or did not open an email prompt and visited HeLP-Diabetes up to 5 days after sending one.

Examining all the user visits (N=918) that were recorded following the sending of each email prompt, from the first one in February 2014 until the last in May 2015, the time taken for users to visit HeLP-Diabetes after an email prompt was sent ranged from the same day to 23 days. The median time taken to visit HeLP-diabetes was 1 day after receiving an email prompt, with an IQR of 0 to 5 days (ie, 25% visited HeLP-diabetes on the same day of receiving an email prompt, and 75% did so up to 5 days of receiving a prompt). The percentage of users who opened or did not open an email and visited HeLP-Diabetes up to 5 days after a prompt was sent is shown in Figure 3. No user visited HeLP-Diabetes within 5 days after the email prompts “How do I lose weight and feel better?,” “Designing your care plan,” and “Shopping for food” were sent.

Data for 3 email prompts (“Keeping your bones healthy,” “HeLP-Diabetes Newsletter 9-Anxiety,” and “HeLP-Diabetes Newsletter 10-Break a sweat this summer!”) were excluded because the time between sending the prompt and the next email prompt was less than 5 days.

The chi-square test identified 19 email prompts (out of the 39 analyzed ones) that showed a statistically significant association (*P*<.05) between opening an email and visiting HeLP-Diabetes up to 5 days after an email prompt was sent (see Table 1). Out of those 19 email prompts, “World Diabetes Day” had the highest percentage of users who opened an email prompt and visited HeLP-Diabetes: the prompt was sent to 308 users, 43.2% (133/308) opened the email and of these 28.6% (38/133) then visited the website. The next most successful prompt was “HeLP-Diabetes Newsletter 12-Your diabetes is in your hands,” which was sent to 233 users and 27% (25/92) of users opened and then visited HeLP-Diabetes. The email prompt “Making HeLP-Diabetes easier” was sent to a larger number of users than the previous 2 prompts (N=428), but it had the lowest response rate; although 40.2% (172/428) opened the email prompt, only 8.1% (14/172) of users then visited HeLP-Diabetes. Starting from the email prompt “HeLP-Diabetes Newsletter 11-Holiday preparations,” the sample was bigger and the email prompts showed a significant association between opening the email prompt and visiting HeLP-Diabetes, with the exception of “HeLP-Diabetes Newsletter 13-Get rid of your medication worries!” and “Autumn health reminder.” These 2 email prompts were sent to a relatively larger number of users compared with earlier email prompts, but they did not have a high percentage of users who opened an email prompt and visited HeLP-Diabetes.

**Table 1:** 

Email prompt title	Users who visited HeLP-Diabetes % (n/N)	Users who opened an email prompt % (n/N)	Users who opened an email prompt and visited HeLP-Diabetes % (n/N)	Chi-square result *X*^2^(df^a^, N), *P* value
How are your New Year's resolutions going?^b^	1 (1/71)	38 (27/71)	0 (0/27)	0.6 (1, 71), .43
HeLP-Diabetes Newsletter 6-Medication^b^	1 (1/69)	27 (19/69)	5 (1/19)	2.7 (1, 69), .10
Boosting your health during winter^b^	3 (3/79)	40 (32/79)	6 (2/32)	0.9 (1, 79), .35
Best diet advice!^b^	2 (2/81)	44 (36/81)	2 (1/36)	0.02 (1, 81), .87
Share your personal experience with us!^b^	2 (2/69)	36 (25/69)	8 (2/25)	3.6 (1, 69), .06
How do I lose weight and feel better?^b^	0 (0/83)	38 (32/83)	0 (0/32)	No user visited HeLP-Diabetes
Designing your care plan^b^	0 (0/90)	38 (35/90)	0 (0/35)	No user visited HeLP-Diabetes
HeLP-Diabetes Newsletter 7-Making changes^b^	5 (5/98)	31 (31/98)	9 (3/31)	2 (1, 98), .16
It's Springtime^b^	3 (3/99)	39 (39/99)	5 (2/39)	1 (1, 99), .32
Happy Easter	2 (2/102)	37 (38/102)	5 (2/38)	3.4 (1, 102), .06
Shopping for food	0 (0/103)	22 (23/103)	0 (0/23)	No user visited HeLP-Diabetes
Achieving your goals^b^	.9 (1/106)	30.2 (32/106)	3 (1/32)	2.3 (1, 106), .13
HeLP-Diabetes Newsletter 8-Personal experiences^b^	2.8 (3/108)	30.6 (33/108)	6 (2/33)	1.9 (1, 108), .17
How many meals do you eat per day?^c^	10.7 (12/112)	41.1 (46/112)	21 (10/46)	9.9 (1, 112), <.001
What you need to know about hypoglycemia!^c^	8.5 (11/130)	40.8 (53/130)	18 (10/53)	12.5 (1, 130), <.001
Are you a complementary therapy user?^c^	6.6 (9/136)	31.6 (43/136)	18 (8/43)	14.6 (1, 136), <.001
Sexual health-let's talk about it!^c^	4.8 (7/145)	27.6 (40/145)	5 (2/40)	0.004 (1,145), .95
Fasting during Ramadan^c^	8.4 (14/167)	28.7 (48/167)	16 (8/48)	6 (1, 167), .01
HeLP-Diabetes Newsletter 11-Holiday preparations	8.8 (16/182)	35.7 (65/182)	18 (12/65)	11.8 (1, 182), .001
How to handle the summer heat?	10.8 (23/213)	36.6 (78/213)	23 (18/78)	19.3 (1, 213), <.001
HeLP-Diabetes Newsletter 12-Your diabetes is in your hands	12.4 (29/233)	39.5 (92/233)	27 (25/92)	30.3 (1, 233), <.001
HeLP-Diabetes Newsletter 13-Get rid of your medication worries!^c^	5.4 (13/242)	36 (87/242)	10 (9/87)	6.6 (1, 242), .01
Smile - You're on Camera!	6.8 (17/249)	35.7 (89/249)	14 (13/89)	13.2 (1, 249), <.001
Autumn health reminder^c^	4.5 (12/268)	35.4 (95/268)	5 (5/95)	0.2 (1, 268), .64
HeLP-Diabetes Newsletter 14-What's happening this October?	11.5 (33/286)	38.8 (111/286)	21.6 (24/111)	18.1 (1, 286), <.001
World Diabetes Day	14.6 (45/308)	43.2 (133/308)	28.6 (38/133)	36.6 (1, 308), <.001
HeLP-Diabetes Newsletter 15-Shopping done the right way	10.5 (35/333)	38.1 (127/333)	22.8 (29/127)	33.2 (1, 333), <.001
HeLP-Diabetes Newsletter 16-Eye care	8.9 (30/338)	42.0 (142/338)	17.6 (25/142)	23.1 (1, 338), <.001
Happy Holidays	6.9 (24/346)	39.9 (138/346)	15.9 (22/138)	28.8 (1, 346), <.001
New Year Tips	8.9 (31/348)	43.1 (150/348)	18.7 (28/150)	30.9 (1, 348), <.001
HeLP-Diabetes Newsletter 17-Change for 2015	8.9 (32/358)	34.9 (125/358)	22.4 (28/125)	42.8 (1, 358), <.001
How to manage your diabetes using the HeLP-Diabetes care plan?	10.6 (40/376)	42 (158/376)	20.3 (32/158)	26.5 (1, 376), <.001
HeLP-Diabetes Newsletter 18-Alcohol, love and activity in February	9.5 (37/390)	41.8 (163/390)	20.2 (33/163 )	37.7 (1, 390), <.001
Get to know HeLP-Diabetes	6.7 (27/404)	44.1 (178/404)	12.9 (23/178)	19.9 (1, 404), <.001
HeLP-Diabetes Newsletter 19-Spring, delicious recipes and dark chocolate	10.3 (42/407)	36.9 (150/407)	22.7 (34/150)	39.1 (1, 407), <.001
What HeLP-Diabetes can do for you….	7.5 (31/416)	38.5 (160/416)	18.1 (29/160)	42.9 (1, 416), <.001
HeLP-Diabetes Newsletter 20-What can you eat?	6.3 (26/416)	38.9 (162/416)	12.3 (20/162)	16.8 (1, 416), <.001
Making HeLP-Diabetes easier	3.5 (15/428)	40.2 (172/428)	8.1 (14/172)	18.3 (1, 428), <.001
HeLP-Diabetes Newsletter 21-Mindfulness, HeLP-Diabetes and fruit sugar	6.7 (29/432)	39.4 (170/432)	15.3 (26/170)	33 (1, 432), <.001

^a^df: degrees of freedom.

^b^2 cells (50.0%) have expected count less than 5.

^c^1 cell (25.0%) has expected count less than 5.

#### Association Between Text Message Prompts and Visits to HeLP-Diabetes

There were 7 text message prompts sent between October 2014 and May 2015. None of these 7 text message prompts showed a statistically significant association between subscribing to receive text message prompts and visiting HeLP-Diabetes (see Table 2).

### Stage 2

Objective 3 was to explore features of specific prompts to understand why they did or did not appear to lead to subsequent visits to HeLP-Diabetes.

#### Participants’ Characteristics

There were 6 patient representatives who agreed to participate. Five out of the 6 representatives had worked with us previously, and they provided regular feedback about prompts’ frequency, content, and timing from early 2014. The sixth participant started working with us in mid-2015 and only provided feedback on 1 prompt. All the participants were over the age of 50 years. Two of them were males, and 4 participants had degree-level qualifications. Most of the participants rated their computer experience as medium to high. The length of diabetes diagnosis ranged between 5 and 40 years (see Table 3).

#### Preference of Email Prompt Content

The main findings from the interviews related to participants’ likes and dislikes of the prompts are presented below, accompanied with illustrative quotes.

##### Likes

Participants preferred short and clear email prompt content with an overview at the beginning of an email summarizing the content. Participants suggested including as many links to HeLP-Diabetes pages and section as possible without overwhelming users. One participant liked it when a clear description of what the links included in a prompt would direct the users to what was provided:

So, someone might say, I haven’t got time to watch a video, and they look at their watch, and next the computer says, watch the 3-minute video, so that would be good, because 3 minutes is nothing, isn’t it? I like that.

P4; email prompt title: HeLP-Diabetes Newsletter 12-Your diabetes is in your hands.

Some participants liked it when the emails started by greeting them with their usernames, whereas others felt that personalizing emails in such a way should only be done in short emails and that newsletters should not include their usernames. One user felt that personalizing emails did not make a big difference to the content and how engaging it was.

**Table 2:** 

Text message prompt topics	Subscribed users who visited HeLP-Diabetes % (n/N)	Unsubscribed users who visited HeLP-Diabetes % (n/N)	Chi-square result *X*^2^(df^a^, N), *P* value
Flu jab reminder	5.8 (10/172)	10.9 (14/129)	2.6 (1, 301), .11
Home exercises	6.5 (12/185)	2.1 (3/142)	3.5 (1, 327), .06
Eating and drinking on holidays	4.0 (8/199)	5.3 (8/151)	0.3 (1, 350), .57
January blues	5.3 (10/190)	4.8 (8/167)	0.04 (1, 357), .83
Sharing problems and advices	4.8 (10/209)	5.6 (9/161)	0.1 (1, 370), .72
National Health Service medical exemption certificate	4.7 (11/234)	3.5 (6/173)	0.4 (1, 407), .53
Specialist and technical support	2.8 (7/246)	2.8 (5/180)	0.002 (1, 426), .96

^a^df: degrees of freedom.

**Table 3:** 

ID	Sex	Age, in years	Education level	Length of diabetes diagnosis, in years	Computer experience	When user joined the team, number of prompts the user provided feedback on
P1	Female	55	Degree	15	High	From mid-2014, 8 prompts
P2	Male	50	Postgraduate	5	High	From mid-2015, 1 prompt
P3	Female	60	Degree	20	Medium-high	From early 2014, 10 prompts
P4	Male	58	Grammar school	12	Medium	From early 2014, 9 prompts
P5	Female	68	Postgraduate	40	Medium-high	From early 2014, 12 prompts
P6	Female	69	A level	10	Medium	From early 2014, 12 prompts

Popular content included the use of empowering statements in the titles or in the body of the email such as in the email prompt “HeLP-Diabetes Newsletter 12-Your diabetes is in your hands,” statements referencing health professionals or linking to their recommendations, email titles written as questions (eg, “How to handle the summer heat?” and “HeLP-Diabetes Newsletter 20-What can you eat?”), and including news or HeLP-Diabetes-related updates were seen as the most attention-grabbing type of content:

Well, obviously new developments and things like that, and research and obviously this October stuff. Things like that, topical things. Topical things to keep people engaged. Just anything new that’s coming out, and keeping people up to date with research and stuff like

P6

As for the visual appeal of email prompts, all the participants favored bold colors for the text, bullet point use, short emails (specifically newsletters) not exceeding A4 in length, and the use of pictures.

##### Dislikes

The majority of participants did not like wordy email prompts with lots of information; they felt that prompts should have information written succinctly and concisely:

I don't like a big sheet where it’s all mangled up together.

P1

Too much info. Too much info. I wouldn’t really be reading that.

P2; email prompt title: HeLP-Diabetes Newsletter 20-What can you eat?

Participants felt prompts should be easily understood and disliked the use of complicated medical terminology or language that was hard to understand.

Most of the participants disapproved of directive advice. They felt, as people living with a chronic condition, that they were told on a daily basis what they should or should not be doing when it comes to any aspect of their type 2 diabetes self-management. They preferred the use of the word “try” rather than “orders” when advice was given:

This is very directive - stay optimistic, stay happy. Rather than, try to stay optimistic is more of an empowering sort of thing.

P2; email prompt title: HeLP-Diabetes Newsletter 12-Your diabetes is in your hands

Perceived irrelevancy of email prompt content was a major reason behind not clicking the links embedded in the prompt. Irrelevant content included specific content targeting small subgroups of participants (eg, content targeting only smokers). Participants preferred more general content that applied to the needs of different patients.

When it came to the visual aspects of email prompts, participants disliked faint colors for text or pictures and particularly disliked it when color of the included links was indistinguishable from the rest of the written content.

## Discussion

This mixed-method study provides an insight into the potential components or characteristics (specifically delivery modes and content) that can promote engagement with a DHI targeting self-management of type 2 diabetes (HeLP-Diabetes) and the factors that may influence their effectiveness.

### Summary of Findings

Just under half of email prompts showed a significant association with visits to HeLP-Diabetes. Nineteen out of the 39 email prompts (49%) showed a significant association with visits to HeLP-Diabetes up to 5 days after an email prompt was sent, whereas none of the text message prompts showed a significant association. Furthermore, 75% of HeLP-Diabetes visits occurred in the 5 days after an email prompt was sent.

The think-aloud interviews suggested that an email prompt should be relevant to recipients, short and concise, easy to understand, with simple language and short sentences, contain links to the intervention, contain nondirective advice and aim for an empowering approach, and contain news and updates. Preferred visual aspects included the use of bullet points, pictures, and bold colors.

### Fit With Literature

The email prompts’ results are consistent with the literature regarding engagement prompts; prompts may promote engagement but have a small to moderate effect as shown in a published meta-analysis [10]. However, text message results were unexpected, as the literature shows that text message prompts are better than emails [19]. This result might be because of an older sample not owning smartphones to click the links, the text messages not being detailed enough to be attention-grabbing or not tailored to a degree that facilitates behavior change.

The results of the study showed that, unlike other published studies [29,30], later prompts were not associated with reduced visits to the DHI. There was no downward trend but rather visits were fluctuating; this fluctuation might be because of the prompt content, frequency, timing, and other possible variables.

Some of the preferred email content identified in the interviews was consistent with what is recommended in the literature; the use of nondirective language in face-to-face health or behavior change-related settings has an influence on behavior change [31]. This type of language was also recommended to be used in DHI content [32]. Another feature is the inclusion of news articles or updates to HeLP-Diabetes sections and pages; one study that examined the effect of email prompt content on engagement showed that the inclusion of links to news items on a DHI showed a positive trend toward engagement [18]. Finally, inclusion of health professional references has been reported to be preferred by patients using a service [33]. Personalizing the content of DHIs is also recommended in the literature; however, it is still unclear what level of tailoring prompts should have [32,34].

### Strengths and Limitations of the Study’s Methodology

There were a number of key strengths of this study, including the use of both quantitative and qualitative methods to achieve the research aim, as the combination of methods complemented and provided a clearer picture of the results. Quantitative data cleaning and validating was conducted and reviewed by 2 authors, along with reviewing the interview transcripts and coding to ensure rigor and transparency. The quantitative outcome measure (ie, HeLP-Diabetes user visits) used in this study was objective, meaningful, highly sensitive, and responsive to change, as opposed to subjective measures such as questionnaires. A final key strength was that this research was conducted in a real-life setting rather than a controlled setting where variables that might influence causation are removed.

The study had some limitations, which is to be expected in the emerging field of DHI engagement prompt research. The first main limitation concerned the prompts; the number of users who received the earlier email prompts was small and underpowered to detect an association between receiving a prompt and visiting HeLP-Diabetes; some email prompts’ opens might have not been accounted for if the embedded images were not downloaded; and only a small number of text messages were sent because of technical reasons. The second limitation concerned the think-aloud participants; 6 participants may not have been enough to identify a larger variety of HeLP-Diabetes users’ possible preference for prompt content, however, in the field of human-computer interaction, using 5 users in a usability study is enough to show 85% of design problems that need to be fixed [35]. In addition, being experienced patient representatives who have helped with developing HeLP-Diabetes and prompts, they might not reflect the type of users who use HeLP-Diabetes. In particular, their demographic characteristics were dissimilar to those of many of the registered users in terms of educational qualifications and computer expertise. Another issue related to patient representatives was the fact that it was not possible to analyze whether they visited HeLP-Diabetes following the prompts that were shown to them. The interviews only provided data about the features they liked or not rather than whether the feature would lead to a visit to HeLP-Diabetes or not. Hence, the data did not allow for exploration of engagement behavior. However, it was not possible to recruit HeLP-Diabetes registered users, so patient representatives were asked to be interviewed. The final limitation was that prompts are context-specific interventions, which is why the results may not be generalizable across other DHI or other types of interventions targeting type 2 diabetes self-management. These limitations show that this study was good for hypothesis generation rather than effectiveness determination.

### Research Implications

This mixed-method study provides a means to explore and optimize the effect of different prompt characteristics on engagement with any DHI before conducting an RCT to determine the effectiveness of the prompts. This type of study can be conducted within any research evaluating a DHI that uses prompts to explore their characteristics.

By mostly selecting prompts that showed a significant association with visiting HeLP-Diabetes for the think-aloud interviews, the study results identified characteristics of email prompts that could be tested in a pilot RCT by the research team. Also, the quantitative stage of the study showed that some emails were associated with visits to HeLP-Diabetes, whereas none of the text messages showed any association. This led to a pilot RCT to assess the effect of different modalities.

One important characteristic that needs to be researched further in future studies is the frequency of prompts. For example, one systematic review that looked at frequency of prompts for DHIs targeting different health behaviors found that high-intensity and low-intensity prompts or irregular prompts both yielded positive engagement [36]. In addition, another study showed that text message prompts were more effective at changing health behaviors if they were sent daily compared with if they were sent less frequently [37]. In this study, the frequency of the prompts was based on the feedback given by patient representatives, because participants were registering over time and no fixed sample was available to test different frequencies. Hence, future studies can explore the frequency of engagement prompts.

Usage data analysis is a challenging area of research, as it involves considering and balancing advantages and disadvantages of using specific measures. In this study, “email opens” was used instead of links clicked because users might be triggered to visit HeLP-Diabetes without clicking the links in the email. Visits to HeLP-Diabetes were used instead of Web page visited, which would have shown users’ interests and whether they visited the pages in the prompts or not. However, as with the earlier measure (ie, links clicked and email opens), visits to HeLP-Diabetes are more general and better able to catch users’ activity than a specific measure. Future studies can compare the results of different measures and investigate which ones can capture engagement with DHI because of receiving prompts accurately.

The relationship between engagement with DHI and health outcome improvement was not the focus of this study. The HeLP-Diabetes RCT [21] conducted a subgroup analysis to assess the association between engagement and improvement in diabetes-related clinical outcome; the results were positive. However, it was not possible to determine whether engagement led to health improvement or vice versa; this issue can be explored in future studies, which can also look at the level of engagement that can lead to health improvement (ie, effective engagement) [14].

The data from stage 2 was not rich enough to facilitate deeper interpretation of how the reactions to the prompts led to subsequent behaviors. This may have been because of the method of data collection—“think-aloud interviews” are a method emanating from human-computer interaction science, mostly used to test usability issues rather than look for deeper meaning. Alternatively, it may be that our participants in stage 2, who had all worked with the HeLP-Diabetes team and were used to contributing feedback and ideas for the program, had become used to providing actionable feedback rather than deeper reflections. It would be useful to recruit naïve users in future studies and perhaps undertake additional interviews after the “think-aloud” data collection to obtain richer data.

### Conclusions

This study showed that specific email prompts were associated with greater engagement with a DHI targeting type 2 diabetes self-management (HeLP-Diabetes), but that was not the case for text messages. Participants tended to open an email prompt and visit the DHI up to 5 days of receiving the prompt. The prompts explored in think-aloud interviews led to the identification of the prompt content features that users liked (eg, new content) or disliked (eg, directive advice). The study results can be explored further in future RCTs evaluating characteristics of engagement prompts.

## Appendix

Multimedia Appendix 1 Prompts date, delivery mode, content, number of recipients.

Multimedia Appendix 2 List of prompts used in the think aloud interviews.

Multimedia Appendix 3 Interview schedule guide.

## Abbreviations

CCG: clinical commissioning group
DF: degrees of freedom
DHI: digital health intervention
HeLP-Diabetes: Healthy Living for People with type 2 Diabetes
IQR: interquartile range
RCT: randomized controlled trial
RR: relative risk
UCL: University College London
