# Supplementary material for: Promoting Engagement With a Digital Health Intervention (HeLP-Diabetes) Using Email and Text Message Prompts: Mixed-Methods Study
Source: Interact J Med Res. 2017 Aug 22;6(2):e14. doi: 10.2196/ijmr.6952 (PMC5566257; doi:10.2196/ijmr.6952)
Supplement: Multimedia Appendix 1 [file ijmr_v6i2e14_app1.pdf]

Prompt modes	Prompt title	Summary of prompt content	Date	No. recipients/ No. total registered users [1,2]
Email	How are your New Year's resolutions going?	The email listed various tips and advice on how to maintain and achieve new year's health-related resolutions.	06/02/2014	71/72
Email	HeLP-Diabetes Newsletter 6-Medication	The newsletter described tools and tips to help with taking medications, video about diabetic patients and their experience with medication, the Sharing Data Saves Lives Campaign and the encouragement to use the Ask the Expert thread.	14/02/2014	69/69
Email	Boosting your health during winter	The email listed advice for boosting health during winter including beating winter blues, flu vaccination and looking after feet health.	20/02/2014	79/79
Email	Best diet advice!	This email introduced the dietician thread in the forum for any diet questions, the practical diet advice page in HeLP-Diabetes and user recommended recipe thread.	27/02/2014	81/81
Email	Share your personal experience with us!	This email encouraged users to share their personal experience of any aspect of Type 2 Diabetes and introduced the people stories section.	07/03/2014	69/69
Email	How do I lose weight and feel better?	This email discussed why losing weight is healthy and who should lose weight, as well as tips on how to do that.	14/03/2014	83/83
Email	Designing your care plan	This email introduced the diabetes care plan tool and how using it helps with managing Type 2 Diabetes.	21/03/2014	90/90
Email	HeLP-Diabetes Newsletter 7-Making changes	The newsletter contained tips for small increases in physical activity, a description of the reminder tools, quick guides and physical activity experience videos of diabetic patients, update on Sharing Data Saves Lives Campaign and a news article about chocolate and red wine.	28/03/2014	98/98
Email	It's Springtime	This email coincided with the beginning of Spring and included tips on exercising in the nice weather and how food can affect mood.	03/04/2014	99/99
Email	Happy Easter	This email described HeLP-Diabetes content related to Easter Holiday such as shopping for food and resisting chocolate, and travel tips for the holiday.	10/04/2014	102/102
Email	Shopping for food	This email focused on food labels and information to consider when shopping for food.	16/04/2014	103/103
Email	Achieving your goals	This email discussed how achieving goals can help with raising confidence, how to achieve goals, and what to do if they are not achieved, including dealing with	24/04/2014	106/106

		emotions.		
Email	HeLP-Diabetes Newsletter 8-Personal experiences	The newsletter focused on personal experiences of diabetic patients and described the personal stories section of HeLP-Diabetes, available resources for family and friends, a forum post of personal experience of gastric band surgery written by one of our users and a news article about how Cognitive Behaviour Therapy can improve outcomes for people with Type 2 Diabetes.	30/04/2014	108/109
Email	How many meals do you eat per day?	This email included recommendations spacing out and choosing meals and healthy snacks.	08/05/2014	112/121
Email	Keeping your bones healthy	This email discussed the importance of Vitamin D and exercises to keep the bones healthy.	16/05/2014	127/129
Email	What you need to know about hypoglycaemia!	This email included advice and information about hypoglycaemia (low blood glucose).	21/05/2014	130/134
Email	HeLP-Diabetes Newsletter 9-Anxiety	The focus of this newsletter was on coping with anxiety, describing the online Cognitive Behavioural Therapy programme and the quiz for 'emotion diagnosis', as well as a news article on exercise protecting from the effects of overeating.	29/05/2014	131/134
Email	Are you a complementary therapy user?	This email discussed alternative therapy.	03/06/2014	136/144
Email	Sexual health-let's talk about it!	This email included advice about sexual health and how diabetes can affect it and what do it if experiencing problems.	20/06/2014	145/150
Email	HeLP-Diabetes Newsletter 10-break a sweat this summer!	The newsletter contained tips for staying motivated with a physical activity routine and exercising in the heat; a reminder tool; a quiz with answers providing personalised tips on maintaining physical activity; and two news articles, one about eating large meals and weight loss, and the other about the link between incretins and pancreatitis.	27/06/2014	167/167
Email	Fasting during Ramadan	This email introduced Ramadan (fasting month for Muslims).	30/06/2014	167/177
Email	HeLP-Diabetes Newsletter 11-Holiday preparations	The newsletter contained tips and things to do before traveling and during the trip; a tool for keeping track of medication; and two news articles: one about an initiative by Diabetes UK, and the other about the NHS rating as a top health service in the developed world.	16/07/2014	182/200
Email	How to handle the summer heat?	The email included tips to avoid heat exhaustion such as exercising in cool locations and keeping hydrated.	04/08/2014	213/224
Email	HeLP-Diabetes Newsletter 12-Your diabetes is in your hands	This newsletter focused on empowering diabetic patients by pointing out that the skills they need to improve their diabetes management, a brief description of	21/08/2014	233/238

		diabetes care plan tool, and a video featuring discussion between health professionals and patients about self-management, and two news articles: one on aerobic and strength training and the other on how Metformin helps people live longer. There was also a Member Spotlight on a user sharing his diet journal on the HeLP-Diabetes forum.		
Email	HeLP-Diabetes Newsletter 13-Get rid of your medication worries!	This newsletter focused on dealing with worries related to receiving new medications specifically and taking medications in general. It included common questions and experts answers, and a news article about NICE considering expansion of criteria relating to acceptance for weight loss surgery.	05/09/2014	242/249
Email	Smile - You're on Camera!	The email focused on oral care tips including using the health profile and medicine list tools to take to the dentist and when to brush the teeth.	17/09/2014	249/260
Email	Autumn health reminder	This email focused getting the flu vaccine.	02/10/2014	268/279
Email	HeLP-Diabetes Newsletter 14-What's happening this October?	This newsletter included tips on how to enjoy special occasions without over-indulging in terms of food and drinks, the smoking alcohol cessation campaigns, HeLP-Diabetes layout update, a news article about low carbohydrate diet and a teaser about a news article for next month's issue.	17/10/2014	286/303
Text message	Flu jab reminder	This text stated: Do you need the flu jab? Visit https://www.help-diabetes.org.uk for more information. If you want to unsubscribe update your profile.	31/10/2014	172/301
Email	World Diabetes day	This email contained the three key messages for World Diabetes Day campaign for 2014 and links to HeLP-Diabetes that facilitate actions to support the campaign.	09/11/2014	308/327
Text message	Home exercises	This text stated: Feeling the cold? Why not warm up with some simple home exercises at https://www.help-diabetes.org.uk/. If you want to unsubscribe update your profile.	24/11/2014	185/327
Email	HeLP-Diabetes Newsletter 15-Shopping done the right way	This newsletter contained tips on planning meals and shopping for food, and tools to help with this, such as for setting goals and using reminders; there was also a news article about a cure for Type 1 Diabetes and an achievement of one of our users reaching his weight loss goal and sharing his journey in the forum.	29/11/2014	333/337
Email	HeLP-Diabetes Newsletter 16-Eye care	This newsletter focused on eye health and included tips for eye care, videos about diabetic patients who experienced eye problems, using the diabetes care plan tool, an	09/12/2014	338/343

		announcement about subscribing to text messages, a guide for quitting smoking and a news article about Metformin.		
Text message	Eating and drinking on holidays	This text stated: Would you like to enjoy your festive meals and drink safely during the holiday period? Read our guide at https://www.help-diabetes.org.uk/. If you want to unsubscribe update your profile.	18/12/2014	199/350
Email	Happy Holidays	This email included tips for enjoying the holidays in terms of eating without feeling guilty, encouraged users to share their recipes, included a link about social life and a teaser for next newsletter.	22/12/2014	346/347
Email	New Year Tips	This email focused on real tips from researcher and author Brian Wansink on slimming down and making being healthy easier.	12/01/2015	348/354
Text message	January blues	This text stated: Are you feeling the January blues? Then visit our happiness and well-being page for steps to overcome this feeling that can affect everyone. https://www.help-diabetes.org.uk/. If you want to unsubscribe update your profile.	20/01/2015	190/357
Email	HeLP-Diabetes Newsletter 17- Change for 2015	This newsletter highlighted how HeLP-Diabetes can assist users who want to make health-related changes as part of their New Year resolutions, a video about the Mediterranean diet and a news article about NICE guidance on weight loss surgery.	27/01/2015	358/358
Text message	Sharing problems and advices	This text stated: A problem shared is a problem halved. Are you confused by the different messages about diabetes? Do people offer different advice? Or can you offer some? Check our FAQs to find out which messages to follow or check our Forum to see what other people advice.	11/02/2015	209/370
Email	How to manage your diabetes using the HeLP-Diabetes care plan?	This email advised on how to use the HeLP-Diabetes care plan in consultations or clinic visits.	18/02/2015	376/388
Email	HeLP-Diabetes Newsletter 18- Alcohol, love and activity in February	The newsletter contained tips to succeed in the Dry January Challenge of quitting alcohol and information about that; and information about support and love from family, friends, local support groups and other types of support available for Type 2 diabetic patients. It also contained a news article about physical activity. It was the first newsletter to include pictures.	27/02/2015	390/402
Text message	NHS medical exemption certificate	This text stated: What do you know about NHS medical exemption certificates? Do you know that there	15/03/2015	234/407

		is a fine of £100 if free prescriptions are claimed without a valid exemption certificate? Read more about it on https://www.help-diabetes.org.uk/living-working-with-diabetes.		
Email	Get to know HeLP-Diabetes	This email quoted a user interviewed about HeLP-Diabetes and how he learned something new from HeLP-Diabetes.	19/03/2015	404/406
Email	HeLP-Diabetes Newsletter 19- Spring, delicious recipes and dark chocolate	The newsletter contained tips for exercises for all levels; tips for outdoor activities; recipe for cookies, scones and fruit kebabs; and a news article about chocolate.	30/03/2015	407/414
Email	What HeLP-Diabetes can do for you....	This email listed why using HeLP-Diabetes can help the user to manage their condition and also included an email address to contact for technical support.	23/04/2015	416/416
Email	HeLP-Diabetes Newsletter 20- What can you eat?	This newsletter focused on tips about having a balanced diet, using a dietician thread and a news article about exercise and reducing waistline.	29/04/2015	416/418
Email	Making HeLP-Diabetes easier	The email included instructions on how to use HeLP-Diabetes, such as logging in.	15/05/2015	428/430
Text message	Specialist and technical support	This text stated: Hello [username]. Visit https://www.help-diabetes.org.uk/forum-help/forum for support from people living with diabetes, diabetes specialists and technical support.	21/05 / 2015	246/426
Email	HeLP-Diabetes Newsletter 21- Mindfulness, HeLP-Diabetes and fruit sugar	The newsletter contained a step-by-step guide on how to use an online course on HeLP-Diabetes, description of an activity booklet and one research article about fruit sugar.	27/05/2015	432/433

[1]No. recipients stands for number of users who subscribed to receive a prompt. No. total registered users stands for number of users in total whether subscribed to receive prompts or not. The denominator changes as new users sign up or unsubscribe to HeLP-Diabetes over the course of the study

[2] There are some inconsistencies in the number of recipients and total registered users due to the many reasons, for example: users deregistered or unsubscribed.
