# Supplementary material for: Promoting Engagement With a Digital Health Intervention (HeLP-Diabetes) Using Email and Text Message Prompts: Mixed-Methods Study
Source: Interact J Med Res. 2017 Aug 22;6(2):e14. doi: 10.2196/ijmr.6952 (PMC5566257; doi:10.2196/ijmr.6952)
Supplement: Multimedia Appendix 2 [file ijmr_v6i2e14_app2.pdf]

How to handle the summer heat?

This email contains graphics, so if you don't see them, view it in your browser

HeLP Diabetes™ Healthy Living for People with type 2 Diabetes

Dear Ghadah,

Summer brings with it sunshine and warmth but sometimes the weather becomes very hot ...

So we wanted to remind you of some tips to help you protect yourself from any complications due to the heat wave…

- Keep hydrated. Maintain your blood glucose levels under control especially in summer as high blood glucose levels (otherwise called hyperglycaemia) lead to urinating more frequently. Make sure you check your blood glucose levels constantly if it is very hot or if you're feeling unwell. To learn how to keep hydrated, visit the *hyperglycaemia* section.
- Exercise in the cool. For exercise examples, visit the *exercise videos* section.
- Look out for signs of heat exhaustion. Signs or symptoms include, sweating a lot, skin feeling clammy, fainting or feeling dizzy, feeling nauseous, rapid heartbeat or headaches. If you experience any of these symptoms, go and rest in a cool place, drink lots of fluids and seek medical care if necessary.
- If you take insulin, make sure you store it in a cool and dry place.

If you have any tips for keeping cool during summer you could share them with the rest of the website members in the *forum*.

Best wishes,
The HeLP-Diabetes Team

HeLP-Diabetes Newsletter 12- Your diabetes is in your hands

Problems reading the newsletter? View it online .

HeLPful NEWS

HeLP Diabetes

Healthy Living for Peop[illegible]
with type 2 Diabetes

Stories, research evidence & ideas for a happier & healthier l[illegible]

Hi Ghadah

August 2014
Studies have shown that people who know more about diabetes and how to manage it have better control of their blood glucose levels and develop fewer complications.

TIP OF THE MONTH

You are the most important person of your diabetes team in managing you[illegible] diabetes.The things you choose to do will have a much bigger effect on you[illegible] diabetes than those anyone else can do for you

There are certain skills that can help you improve your diabetes management.

- understand exactly what diabetes is and how it can affect you. To read more abo[illegible] this, visit the section on understanding diabetes.
- stay optimistic and have confidence in your ability to manage your diabetes. Lea[illegible] more by visiting our happiness and well-being and confidence pages.
- try to work through negative emotions such as guilt or denial. The pages on guilt and denial provide you with tools and tips that can help you manage these emotions.
- don't be afraid to ask for support when you need it from others; whether that be [illegible] family member, friend, health professional or someone else with diabetes. To rea[illegible] more about this, take a look at our support newsletter.

Tool

Have you used the diabetes care plan ?

The care plan provide you with lots of tools to manage your diabetes including, a tool [illegible] help you keep track of your appointments, set goals and plans to manage your diabet[illegible] and a list of your yearly medical reviews.

Give it a go and tell us what you think in the forum or by replying to the newsletter.

Video

You may find it useful to watch the three minutes video on learning to self-manage you[illegible] diabetes in the what is self-management section.

Here you will find discussions between people living with diabetes and doctors on the most important aspects of self-management.

NEWS

Combining aerobic and strength training is best for your blood glucose levels

- A study found that aerobic and resistance training were both useful in reducing HbA1c in a population of people with type 2 diabetes.
- Looked at individually, aerobic training appeared to be the more effective of the t types of training.
- However, the study found that, compared with aerobic or resistance training, combining the two types of training resulted in improvements in their control of th blood glucose levels.

To read more about this study, visit the news and research section

NEWS

Can Metformin help people live longer?

- Researchers looked at a very large sample of UK patient data and compared rat of survival between two groups of people living with diabetes type 2 (those on metformin or sulphonylureas) and carefully-matched patients who did not have t 2 diabetes.
- Surprisingly, the type 2 diabetes group who were given metformin appear to hav rate of survival which is "at least as good" as the matched patients who did not have type 2 diabetes.

To learn more about what this study means for you if you are taking metformin or sulphonylureas, visit the news and research section

Member spotlight

One of our users has been very pro-active in looking after his diabetes, and has recorded his food intake and weight loss every day in the forum. He has lost an impress 19lb and his blood glucose levels are mostly within the normal range. Well done!

Take a look at his journal and share your thoughts and experiences in the forum.

TELL US WHAT YOU THINK

We'd love to hear your views on our newsletter. If you have any suggestions on how w could improve it, or any ideas for future editions.

Please email us at help-diabetes@ucl.ac.uk or leave us a message in our forum. We l forward to hearing from you.

Best wishes
The HeLP-Diabetes team.

Whittington Health NHS UCL

HeLP-Diabetes is a research team at University College London (UCL) that has been funded by the government to create a self-management programme specifically for peo with type 2 diabetes. The aim of the programme is to help people look after themselves take control of their diabetes so they can live healthier and happier. Read more about th HeLP-Diabetes team.

Unsubscribe from this newsletter

Problems reading the newsletter? View it online .

HeLPful NEWS

HeLP Diabetes

Healthy Living for Peop
with type 2 Diabetes

Stories, research evidence & ideas for a happier & healthier li

Hi Ghadah

September 2014
We often hear about or get offered new medications. This can make us feel anxious for several reasons.
This newsletter will explore such concerns and introduce some tools that you might find useful for dealing with any worries you might have about taking your medicines.

TIP OF THE MONTH

Recognising your concerns is the first step to controlling your anxiety.

Some of the common questions about medications include the following:

Will I ever be able to stop treatment?

Sometimes it is possible to stop or reduce your dose of diabetes-related medicines; fo
example, if you lose a substantial amount of weight or get much fitter. We also know th
living healthily can delay the need for medication.

However, diabetes is a long-term condition, which means that most people eventually take several medicines to help manage their diabetes and their overall risk most effectively.

**I have heard worrying or conflicting reports about my medication in the news an
don't know what to do?**

Before deciding to make any changes to your medication dose or stop taking it, seek medical advice and discuss your concerns with your doctor or your diabetes care tean
as some news articles will not be applicable to you.

For example, some news articles report studies that might not have come to a conclus
yet or those with results that apply to very specific groups of people.

To look at examples of news and research done on diabetes and medication, visit the news and research section.

If you can relate to any of these concerns, visit our page on concerns about taking medication where you'll find questions about taking medications and advice on what y
can do to reduce your worry about taking them.

Also, If you have questions about specific medications such as how to take them; wha
the side effects are; can you drive while taking them; take a look at the list of medicatio
categories on the right-hand side of the medicines section and choose the category you're interested in.

For example if your questions are about metformin (a type of medication used for bloo
glucose control), visit the glucose control page and click metformin .

Diabetes Newsletter 13- Get rid of your medication worries!

Tool

Do you have trouble remembering to take your medication? If so it might help to desig
'medicine plan' to keep track of your medicine doses and when to take them.

Visit the medicine planner and start recording your medication to help you keep track
those which help control blood glucose, blood pressure and cholesterol.

Video

Many people living with diabetes type 2 are prescribed oral medication to control their
blood glucose, blood pressure and cholesterol levels.

In the people's stories section, you will find people sharing their stories of how they
started taking them and the side effects they experienced and how they managed tho
effects.

NEWS

NICE considers expansion of weight loss surgery

- New draft guidelines from the National Institute for Health and Care Excellence (NICE) recommends that people with recently diagnosed type 2 diabetes with a [illegible] of 35 or more can be offered an assessment for bariatric surgery.
- Those who might be at particular risk of diabetic complications, such as people [illegible] Asian origin, might be assessed if they have a lower BMI.

To learn more, visit the news and research section

TELL US WHAT YOU THINK

We'd love to hear what you think of our newsletter, if you have any ideas on how we could make it better or any suggestions for future editions, please email us at help-diabetes@ucl.ac.uk or leave a message in our forum. We look forward to hearing from you.

Best wishes
The HeLP-Diabetes team.

Whittington Health NHS UCL

HeLP-Diabetes is a research team at University College London (UCL) that has been funded by the government to create a self-management programme specifically for peo
with type 2 diabetes. The aim of the programme is to help people look after themselves
take control of their diabetes so they can live healthier and happier. Read more about th
HeLP-Diabetes team.

Unsubscribe from this newsletter

Autumn health reminder

This email contains graphics, so if you don't see them, view it in your browser

HeLP Diabetes™ Healthy Living for People with type 2 Diabetes

Dear Ghadah,

Autumn has arrived. It's time to embrace leafy city strolls and country getaways but don't forget autumn is also the start of the flu season…

You can help prevent getting flu this autumn by contacting your GP practice to check if you can get a free flu jab (otherwise known as a flu vaccine).

It is recommended by the Department of Health that everyone with diabetes should have a flu vaccine annually.

Even if you had a flu vaccine last year, you will need another this year because the virus is constantly changing.

Sometimes people worry that having the vaccine will give them the flu. This is not possible because the vaccine consists of a "killed virus" meaning the flu virus is dead before it's put into the vaccine. The virus doesn't make you ill; instead it teaches your immune system how to recognise the live flu virus and how to fight the live virus if it does find its way into your system.

If you'd like to read more about flu and diabetes, please visit the *flu vaccine* page.

Also if you have any tips for preventing coughs and colds, share them with the rest of us in the *forum*!

Best wishes,
The HeLP-Diabetes Team

Not interested any more? *Unsubscribe*

HeLP-Diabetes Newsletter 14- What's happening this October?

World

Problems reading the newsletter? View it online .

HeLPful NEWS

HeLP Diabetes

Healthy Living for Peop
with type 2 Diabetes

Stories, research evidence & ideas for a happier & healthier li

Hi Ghadah

October 2014
October is here and lots of things are happening this month: we're saying a fond farewell to summer and looking forward to autumn with celebrations and campaigns to kick start some healthy habits

TIP OF THE MONTH

How to enjoy special occasions without over-indulging

There are many reasons to celebrate this autumn, including Halloween, Harvest Festiv
Diwali, Yom Kippur, Eid and many others...

Over-indulgence on special occasions can happen to anyone so here are some tips to help you enjoy guilt-free fun….

- Plan ahead; you could ask your host what kind of food they plan to serve and make your food choice ahead of time.
- Try not to attend events when you're hungry - eat something beforehand. Try not to starve yourself in order to over-indulge later because if you arrive hungry, you a
more likely to overeat.
- Be careful with buffets. Decide on what you are going to have and resist the tempta
for second helpings.

For more tips, visit the eating at celebrations page.

Tools

Two major public health campaigns are launched in October; one to encourage smoke
to stop smoking-'Stoptober'- and the other one; 'Sober October' is to help people stay sober throughout October!

To help those of you who would like to join these campaigns, have a look at our page smoking, and visit the alcohol section to design a personalised plan for cutting down c
alcohol.

A new look for HeLP-Diabetes

We have just updated the website based on your really helpful feedback. We hope thi
makes it easier for you to find your way around the website and discover new things.

What do you think of the changes?

Have a look and tell us what you think in the forum.

Diabetes day

This email contains graphics, so if you don't see them, view it in your browser

Healthy Living for People with type 2 Diabetes

Dear Ghadah,

Friday the 14th of November is International World Diabetes Day and this year's theme is about healthy living and diabetes.

The three key messages of the campaign are:

- Make healthy food the easy choice- visit the *practical diet advice* section to find out more.
- Make the right choices - To be able to do this it helps if you understand your food. Visit our *understanding food* section to see how different food and drink influence our bodies.
- Healthy eating begins at breakfast time - do you have any special breakfast recipes? Please share them with us in the *forum.*

Best wishes,
HeLP-Diabetes team

Not interested any more? *Unsubscribe*

This email contains graphics, so if you don't see them, view it in your browser

HeLP Diabetes 2 — Healthy Living for People with type 2 Diabetes

Dear Ghadah,

We talked to one of our users about what new things they learned through HeLP-Diabetes and this is what one of our users found out:

“There was one thing that was important, that I just noticed, on driving, one thing I didn't know, and really ought to have known. If one was diagnosed with Type 2 Diabetes, you tell your motor insurance. And now I've done that.”

To find out more about this issue, visit the *driving* page. You can also find more information about other aspects of life and work in the *living and working with diabetes* section that is updated by health professionals regularly.

Did you find out something new through HeLP-Diabetes, tell us what it is so we can share it with the rest of the users.

Please email us on *help-diabetes@ucl.ac.uk* or post about it in the *forum*.

Best wishes,
HeLP-Diabetes team

Not interested any more? *Unsubscribe*

HeLP-Diabetes Newsletter 20- What can you eat?

5. Try and avoid foods or drinks that are high in fat and / or sugar, such as fizzy drinks, cakes and sweets. These foods can be enjoyed occasionally as part of a healthy balanced diet, but try not to have them too often.

The eatwell plate can help you to decide on the proportions of different types of food to give you the vitamins and minerals you need.

But depending on your individual requirements, you may want to adjust the proportions - for example if you are trying to lose weight or lower your blood glucose levels, reduce the amount of carbohydrate and increase the amount of vegetables on your plate.

There are other types of diet that you might like to try - like a Mediterranean diet, low carbohydrate diet or low calorie diets.

We have added a new page on the website looking at the evidence for these different diets.

We also have very exciting news!

An experienced dietician just joined the HeLP-Diabetes team and she will be very happy to answer any questions you have about food and eating. Or give you suggestions for healthy and tasty recipes.

All you have to do is visit the questions I would like to ask a dietician thread in the forum and post your questions there.

NEWS- Exercise of any kind can reduce waistlines

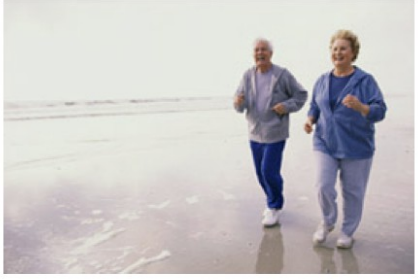

What did the study find?

- Regular low-intensity exercise might be as good as high intensity in helping obese people lose weight.
- All of the exercise groups reduced their weight compared with the no exercise control group.
- On average, all groups showed the same changes in waist circumference:– 4.6 cm at 24 weeks.

Visit the news & research for more information about what this study means to you.

TELL US WHAT YOU THINK

We'd love to hear what you think of our newsletter, if you have any ideas

on how we could make it better or any suggestions for future editions.

Please email us at help-diabetes@ucl.ac.uk or leave a message in our forum. We look forward to hearing from you.

Best wishes
The HeLP-Diabetes team.

HeLP-Diabetes is a research team at University College London (UCL) that has been funded by the government to create a self-management programme specifically for people with type 2 diabetes. The aim of the programme is to help people look after themselves and take control of their diabetes so they can live healthier and happier. Read more about the HeLP-Diabetes team.

Unsubscribe from this newsletter

Making HeLP-Diabetes easier

This email contains graphics, so if you don't see them, view it in your browser

Dear Ghadah,

HeLP-Diabetes is a website with the latest research about type 2 diabetes. You can also find answers to any questions you have about type 2 diabetes and it can help you manage your condition.

We want you to benefit as much as possible from using HeLP-Diabetes so here are some tips to get you going.

1. to protect your personal information *HeLP-Diabetes* needs a username and password to login. You can also login by using your email address as your username. If you have forgotten your password, go to the *lost password* page.

2. once you enter your username and password, you can visit any page, watch any video and use any tool on HeLP-Diabetes. For example, you can start with general information from the *Quick Guides* in the *Understanding diabetes* section. Or for specific information on treatment options for type 2 diabetes – including newer treatments like SGLT2 inhibitors and gliptins - visit the *Treating diabetes* section.

3. if you have a problem with watching the videos visit the *FAQs: Using HeLP-Diabetes* page which has instructions on how to install Flash player. If you have other question not included on this page, post them in the *forum*.

4. most importantly, choose a time and place where you can browse HeLP-Diabetes at your leisure. We're sure you'll find information you didn't know before - some of our users told us they weren't aware they had to notify their *motor insurance* company of their diabetes. Or you might find tools that really help - one of our users told us they loved sending free text reminders to do some simple exercises to their phone using the *my reminders* tool.

We really hope that you find using HeLP-Diabetes easy and worthwhile, but if you have any suggestions to improve it, don't hesitate to post them in the *forum*.

Best wishes,
HeLP-Diabetes team

Not interested any more? *Unsubscribe*

HeLPful NEWS

HeLP Diabetes 2™

Healthy Living for People with type 2 Diabetes

Stories, research evidence & ideas for a happier & healthier life

April 2015

Hello Ghadah Alkhaldi,

Do you constantly ask yourself what can you eat?
Well, let us help you find food that you enjoy *and* keeps you healthy.

A Balanced Diet

The eatwell plate is a good place to start. To make sure you get the right balance of the 5 different food groups try to eat:

1. A variety of fruit and vegetables each day, including pulses. They are rich in vitamins and minerals, high in fibre and low in fat.

2. Some milk and dairy products. If you are trying to cut down on fat, consider choosing lower-fat milk and dairy products.

3. Some meat, fish, eggs, beans and other non-dairy sources of protein. Meat can be high in saturated fat that raises cholesterol levels so choose leaner cuts of meat and remove visible fat from meat and skin from poultry before cooking.

4. Wholegrain starchy carbohydrates, as they are high in fibre - for example try granary, seeded or rye bread instead of white bread, or brown rice instead of white rice. Beans and pulses are also a high fibre source of carbohydrate and offer a healthy alternative to potatoes, rice and pasta.
