# Supplementary material for: Promoting Engagement With a Digital Health Intervention (HeLP-Diabetes) Using Email and Text Message Prompts: Mixed-Methods Study
Source: Interact J Med Res. 2017 Aug 22;6(2):e14. doi: 10.2196/ijmr.6952 (PMC5566257; doi:10.2196/ijmr.6952)
Supplement: Multimedia Appendix 3 [file ijmr_v6i2e14_app3.pdf]

# Email prompts to promote engagement with HeLP-Diabetes: patient representatives' perspective

Interview schedule- HeLP-Diabetes patient representatives

Date and time:
Location:
Interviewer name:
Participant ID number:

Pre-interview checks:
Recorder, mic, mobile phone, security check (notifying someone about the location and time of the interview and how long it will take), paper copy of background information, laptop (with internet access) to show email prompts and £20 voucher as a token of appreciation.

Introduction:

"Thank you very much for taking part in this study. I am Ghadah. You have been an extremely helpful and dedicated member of the patient representative group and that's why we've selected you for an interview. We are interested in what you think about some email prompts (that we will show you on the laptop) that encourage you and other users to visit the website more. What you say will help us tremendously in improving HeLP-Diabetes email prompts so your opinion is very valuable to us. While you are going through the email prompts on the laptop, please say your thought aloud, even if you feel they are not important or negative. Please feel free to say whatever comes into your mind, there are no right or wrong answers. We really want to know your first impression of the email prompts, what you like, what you do not like and how you think it can be changed or improved.

Before we start, would you mind taking another look at the information sheet and sign the consent form? [Present extra copy if needed of information sheet and consent forms]

I would just like to remind you that you are free to stop at any time and if you do not want to answer certain questions then you do not have to. Do you have any questions before we start?

I may take some points during the interview so I can refer to them later, is it ok if I do that?

I am going to start recording now.

Thoughts and feelings about email prompts:

During the interview:

Prompts, if needed (may depend on which email prompt is being viewed):

What is your first impression of the email?

What are you thinking now?

What did you think of the content?

What did you think of the wording used?

What do you think of the website links mentioned in the email prompt?

What did you like/dislike about the prompt?

Before we move to the next one, what do you feel about this prompt overall?

Towards the end of the interview:

Which prompts did you like the most/least? Why?

What was it like to view the email prompts?

Message to HeLP-Diabetes team and other users:

In the end, is there anything you would like to tell the developers or other users of HeLP-Diabetes?

Thank you very much for you time and valuable feedback.

Before we conclude this interview, can you give me some background information?

Background information

Age __________
Sex__________
Education level ____________
How long did you have diabetes? _________________
Experience with computers (high, low, average)

Please accept this £20 voucher as thank you gift.
